# 2021年中国慢性髓性白血病患者关注的问题调查与分析

**DOI:** 10.3760/cma.j.issn.0253-2727.2022.09.008

**Published:** 2022-09

**Authors:** 昱婷 刘, 小帅 张, 悦 侯, 倩 江

**Affiliations:** 北京大学人民医院、北京大学血液病研究所、国家血液系统疾病临床医学研究中心，北京 100044 Peking University People's Hospital, Peking University Institute of Hematology, National Clinical Research Center for Hematologic Disease, Beijing 100044, China

**Keywords:** 白血病，髓样，慢性, 酪氨酸激酶抑制剂, 患者关注问题, 问卷调查, Chronic myeloid leukemia（CML）, Tyrosine kinase inhibitor（TKI）, Patients', concerns, Questionnaire investigation

## Abstract

**目的:**

调查中国成人慢性髓性白血病（CML）慢性期患者在酪氨酸激酶抑制剂（TKI）治疗中关注的问题。

**方法:**

北京大学人民医院于2021年8月至9月，以电子调研问卷的形式对全国范围内的CML患者进行问题关注度的横断面调查，并与2015–2016年调查结果对比分析。

**结果:**

952份问卷可供分析。前5个最受关注的问题依次为“TKI不良反应及处理”（66％）、“TKI停药”（46％）、“CML疾病危险度”（46％）、“TKI药物减量”（42％）和“生活注意事项”（41％）。与2015–2016年相比，本次调查CML患者对“TKI不良反应及处理”、“基因监测”和“化验报告单解读”的关注度显著提高（*P*值均<0.01），对“TKI医保报销政策”、“药物价格”和仿制药相关问题的关注度显著降低（*P*值均<0.01）。多因素分析显示，女性（*OR*＝1.8，95％*CI* 1.4～2.5，*P*<0.001）、年龄大（*OR*＝1.0，95％*CI* 1.0～1.0，*P*<0.001）和学历≥本科（*OR*＝1.8，95％*CI* 1.3～2.4，*P*<0.001）的患者更关注“TKI药物减量”，学历≥本科（*OR*＝1.6，95％*CI* 1.2～2.2，*P*＝0.002）的患者较多关注“CML疾病危险度”，目前服用二代或三代TKI（*OR*＝1.9，95％*CI* 1.3～2.6，*P*<0.001）的患者较多关注“TKI耐药”。

**结论:**

中国CML患者关注最多的是“TKI不良反应及处理”、“TKI停药”、“CML疾病危险度”、“TKI药物减量”和“生活注意事项”。患者关注的问题与其社会人口学因素及疾病治疗现状相关。

## Abstract (en)

**Objective:**

To investigate the concerns of adult patients with chronic myeloid leukemia（CML）in the chronic phase receiving tyrosine kinase inhibitor（TKI）therapy in China.

**Methods:**

A cross-sectional questionnaire including 23 issues of concern was filled by patients with CML nationwide from August to September 2021. The results were compared with those from 2015 to 2016.

**Results:**

Data from 952 questionnaires were analyzed. The five most concerned issues were “TKI-related adverse effects and management”（66％）, “stopping TKI therapy”（46％）, “CML risk assessment”（46％）, “TKI dose reduction”（42％）, and “restrictions in daily life activities”（41％）. Compared with the results from 2015 to 2016, patients paid more attention to “TKI-related adverse effects and management”, “monitoring”, and “interpretation of laboratory reports”（all *P*<0.01）. Concerns of “TKI reimbursement policies”,“price reduction of TKIs”, and issues related to generic TKIs decreased significantly（all *P*<0.01）. Multivariate analysis showed that female patients（*OR*＝1.8, 95％ *CI* 1.4–2.5, *P*<0.001）, elderly patients（*OR*＝1.0, 95％ *CI* 1.0–1.0, *P*<0.001）, or patients with bachelor's degree or higher（*OR*＝1.8, 95％ *CI* 1.3–2.4, *P*<0.001）were more concerned with “TKI dose reduction” than others. Patients with a bachelor's degree or higher（*OR*＝1.6, 95％ *CI* 1.2–2.2, *P*＝0.002）paid more attention to “CML risk assessment”, whereas those currently receiving a second- or third-generation TKI therapy（*OR*＝1.9, 95％ *CI* 1.3–2.6, *P*<0.001）were more concerned about “TKI resistance”.

**Conclusion:**

Patients with CML paid the most attention to “TKI-related adverse effects and management”, “stopping TKI therapy”, “CML risk assessment”, “TKI dose reduction”, and “restrictions in daily life activities”. Patients' sociodemographic covariates and treatment status were associated with their concerns.

2000年后，酪氨酸激酶抑制剂（TKI）显著改善了慢性髓性白血病（CML）患者的生存。国内外多项研究表明，社会人口学特征及社会经济地位与CML患者的治疗选择、服药依从性、监测状况、治疗反应和结局显著相关[1]–[6]。10年前，中国可及的TKI种类少、价格高、绝大多数药物医保政策不能覆盖，导致药物可及性低、患者治疗依从性差、治疗结果不乐观。2015–2016年，我们曾在全国范围内开展调研，结果显示CML患者高度关注“TKI医保报销政策”、“TKI新药进展”及“TKI药物价格”[7]。随着2013年后国产仿制药伊马替尼、达沙替尼等上市，2017年后TKI陆续被纳入全国医保目录、药物价格大幅下降、数种TKI新药临床试验在国内开展，我国CML患者TKI治疗的可及性和药物选择性大大提高[8]–[9]，患者关注的问题也随之改变。了解CML患者在治疗过程中关注的问题，有助于帮助医务工作者了解患者治疗中的需求和困难，从而更有针对性地优化医疗决策，加强医患沟通，提高医疗服务质量，最终改善患者整体治疗结局[10]。目前，国内外少有基于患者视角进行相关分析的数据。本研究对2021年成人慢性期CML患者TKI治疗过程中关注的问题进行横断面调研，并与2015–2016年调查结果比较，分析社会经济因素、医保政策和新药研发给CML患者关注的问题带来的变化及其影响因素，为CML全程管理和患者教育提供针对性的策略。

## 病例与方法

一、研究设计

本研究为非干预性、横断面研究，由北京大学人民医院血液科江倩教授设计和实施。2021年8月至9月，通过微信平台向全国范围内的CML患者发放调查问卷，患者来源于北京大学人民医院血液科江倩教授的CML患者微信群。2015–2016年的调查数据来自2015年9月至2016年9月由北京大学人民医院和北京新阳光慈善基金会共同开展的“慢粒患者生活质量调查”[7]。本研究获得北京大学人民医院伦理委员会的批准（批件号：2020PHB168-01）。

二、调研问卷

问卷包含两个部分，共计10个问题。第一部分包括受访者人口学特征（姓名、性别、年龄、户籍、婚姻状况、学历）以及TKI治疗临床信息（开始TKI治疗距诊断时间、TKI治疗时间、目前和曾经服用的TKI种类、治疗反应）。第二部分包括目前治疗过程中关注的问题，共涉及23个问题，覆盖了2015–2016年的调查中的16个问题，即“TKI医保报销政策”、“TKI新药进展”、“TKI药物价格”、“TKI远期疗效”、“CML基础知识”、“国产与进口TKI转换”、“国产TKI仿制药质量”、“TKI不良反应及处理”、“生育”（在<50岁的女性患者中调查）、“化验报告单解读”、“基因监测”、“TKI停药”、“生活注意事项”、“药物相互作用”、“治疗选择”、“TKI剂量调整”，以及新增加的7个问题：“TKI药物减量”、“CML疾病危险度”、“新冠疫苗”、“TKI耐药”、“病友治疗经验”、“服药依从性”、“一代与二代TKI转换”。问卷链接：https://www.wjx.cn/vm/mB3Vh9B.aspx。

三、统计学处理

患者人口学特征及关注的问题采用描述性统计分析，分类变量采用例数（比例），连续变量采用中位数（范围）描述，组间比较使用Pearson卡方检验、Wilcoxon符号秩检验、Mann-Whitney *U*检验。单因素分析*P*<0.2的变量纳入二元Logistic回归模型进行多因素分析。*P*<0.05为差异有统计学意义。采用SPSS 26.0软件进行统计分析。

## 结果

2021年8月至2021年9月，共收集来自中国30个省、自治区和直辖市的共1365份问卷，其中413份因重要信息缺失（183份）、受访者年龄<18岁（145份）、非慢性期（49份）、TKI治疗时间<3个月（35份）和从未使用TKI治疗（1份）而被删除，最终952份有效问卷被纳入本研究进行分析。

一、受访者特征

本次调查的952例可评估的受访者特征见表1。男533例（56.0％），中位年龄47（18～81）岁，城镇户籍649例（68.2％），已婚762例（80.0％），学历≥本科393例（41.3％）。687例（72.2％）受访者在诊断后6个月内开始TKI治疗，TKI治疗中位时间为58（1～245）个月。受访者填写问卷时，服用伊马替尼499例（52.4％），二代或三代TKI 425例（44.6％），进口药524例（55.0％），国产药400例（42.0％），停药者28例（2.9％）。根据受访者自我报告，治疗反应获得完全细胞遗传学反应（CCyR）者826例（86.8％）、主要分子学反应（MMR）者755例（79.3％）、微小残留白血病未测得（UMRD）者429例（45.1％）。

**表1:** 2021年与2015–2016年慢性髓性白血病慢性期受访者特征比较

特征	2021年（952例）	2015–2016年（1518例）	统计量	*P*值
男性［例（％）］	533（56.0）	939（61.9）	8.373	0.004
年龄［岁，*M*（范围）］	47（18~81）	42（18~88）	9.493	<0.001
户籍［例（％）］			7.374	0.025
城镇	649（68.2）	1046（68.9）		
农村	303（31.8）	461（30.4）		
未知	0（0）	11（0.7）		
婚姻状况［例（％）］			5.347	0.148
未婚	142（14.9）	245（16.1）		
已婚	762（80.0）	1179（77.7）		
离异或丧偶	48（5.0）	88（5.8）		
未知	0（0）	6（0.4）		
学历［例（％）］			29.507	<0.001
<本科	559（58.7）	737（48.6）		
≥本科	393（41.3）	770（50.7）		
未知	0（0）	11（0.7）		
诊断至开始TKI治疗时间［例（％）］			279.148	<0.001
<6个月	687（72.2）	1223（80.6）		
6个月以上	108（11.3）	295（19.4）		
未知	157（16.5）	0（0）		
TKI治疗时间［月，*M*（范围）］	58（1~245）	27（3~83）	20.547	<0.001
目前治疗［例（％）］			110.448	<0.001
伊马替尼	499（52.4）	1101（72.5）		
二代或三代TKI ^a^	425（44.6）	406（26.7）		
其他	28（2.9）^b^	11（0.7）		
目前TKI［例（％）］			68.404	<0.001
进口药	524（55.0）	1009（66.5）		
国产药	400（42.0）	509（33.5）		
其他	28（2.9）^b^	0（0）		
治疗反应［例（％）］			82.180	<0.001
未达CCyR	103（10.8）	381（25.1）		
CCyR未达UMRD	397（41.7）	521（34.3）		
UMRD	429（45.1）	560（36.9）		
未知	23（2.4）	56（3.7）		

注：TKI：酪氨酸激酶抑制剂；CCyR：完全细胞遗传学反应；UMRD：微小残留白血病未测得。^a^ 包括达沙替尼、尼洛替尼、氟马替尼、拉多替尼、普纳替尼、耐克替尼；^b^包括曾服用TKI治疗，目前服用羟基脲、干扰素及停药患者

2015–2016年调查的受访者特征见表1。与2015–2016年受访者相比，本次调查受访者男性比例更低（*P*<0.001），年龄更大（*P*<0.001），农村户籍比例更高（*P*＝0.025），学历<本科比例更高（*P*<0.001）。在TKI治疗中，2021年受访者TKI治疗总时间更长（*P*<0.001），目前服用二代或三代TKI比例更高（*P*<0.001），目前服用国产TKI比例更高（*P*<0.001），获得CCyR及UMRD患者比例更高（*P*<0.001）。

二、患者关注的问题及其影响因素

本次调查中，患者关注问题见图1，关注度超过30％的问题依次为“TKI不良反应及处理”（66％）、“TKI停药”（46％）、“CML疾病危险度”（46％）、“TKI药物减量”（42％）、“生活注意事项”（41％）、“新冠疫苗”（40％）、“TKI医保报销政策”（37％）、“TKI新药进展”（37％）、“药物相互作用”（33％）、“TKI远期疗效”（32％）、“基因监测”（32％）和“TKI耐药”（30％）。与2015–2016年调查共同涉及的16个问题中，受访者对“TKI医保报销政策”、“TKI新药进展”、“TKI远期疗效”、“TKI药物价格”、“CML基础知识”、“生育”、“国产TKI仿制药质量”和“国产与进口TKI转换”的关注度显著降低（*P*值均<0.01）；对“TKI不良反应及处理”、“基因监测”和“化验报告单解读”的关注度显著提高（*P*值均<0.01）；对“TKI停药”、“生活注意事项”、“药物相互作用”、“治疗选择”和“TKI剂量调整”的关注度差异无统计学意义。

**图1:**
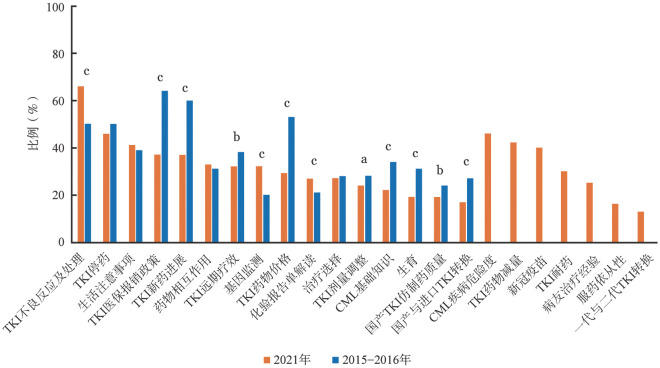
2021年与2015–2016年慢性髓性白血病（CML）患者关注问题的比较 TKI：酪氨酸激酶抑制剂；生育在<50岁的女性患者中调查；^a^*P*<0.05，^b^*P*<0.01，^c^*P*<0.001

在信息完整的患者（779例）中，纳入社会人口学特征及TKI临床治疗信息，对本次调查新增问题中患者关注较多的4个问题的影响因素进行多因素分析。女性（*OR*＝1.8，95％*CI* 1.4～2.5，*P*<0.001）、年龄大（*OR*＝1.0，95％*CI* 1.0～1.0，*P*<0.001）和学历≥本科（*OR*＝1.8，95％*CI* 1.3～2.4，*P*<0.001）的患者更关注“TKI药物减量”，而目前服用二代或三代TKI（*OR*＝0.6，95％ *CI* 0.4～0.8，*P*<0.001）、停药（*OR*＝0.2，95％ *CI* 0.1～0.5，*P*＝0.002）以及目前服用国产TKI（*OR*＝0.7，95％ *CI* 0.5～1.0，*P*＝0.037）的患者关注较少。学历≥本科（*OR*＝1.6，95％*CI* 1.2～2.2，*P*＝0.002）的患者较多关注“疾病危险度”，而治疗反应≥CCyR（*OR*＝0.3～0.6，*P*<0.001～0.028）以及TKI治疗时间长（*OR*＝1.0，95％ *CI* 1.0～1.0，*P*＝0.038）的患者关注较少。治疗反应≥MMR（*OR*＝2.0～2.3，*P*＝0.003～0.006）的患者较多关注“新冠疫苗”，而目前服用二代或三代TKI（*OR*＝0.7，95％ *CI* 0.5～0.9，*P*＝0.010）、停药（*OR*＝0.4，95％ *CI* 0.1～1.0，*P*＝0.050）以及目前服用国产TKI（*OR*＝0.7，95％ *CI* 0.5～0.9，*P*＝0.014）的患者关注较少。目前服用二代或三代TKI（*OR*＝1.9，95％ *CI* 1.3～2.6，*P*<0.001）的患者较多关注“TKI耐药”，而治疗反应≥CCyR（*OR*＝0.3～0.5，*P*<0.001～0.024）的患者关注较少。

## 讨论

本研究是2021年在中国CML患者中进行的大型调研。调研结果显示，目前CML患者最关注的5个问题是“TKI不良反应及处理”（66％）、“TKI停药”（46％）、“CML疾病危险度”（46％）、“TKI药物减量”（42％）和“生活注意事项”（41％）。与2015–2016年相比，本次调研患者更多关注“TKI不良反应及处理”、“基因监测”和“化验报告单解读”；更少关注“TKI医保报销政策”、“TKI新药进展”、“TKI远期疗效”、“TKI药物价格”、“CML基础知识”、“生育”、“国产TKI仿制药质量”和“国产与进口TKI转换”。患者关注的问题与其社会人口学特征及TKI临床治疗特征显著相关。

经济负担曾是CML患者获得最佳治疗的一大阻碍，且经济负担显著影响着患者的TKI治疗选择、服药依从性及疾病监测状况[11]–[12]。因此，不难理解2015–2016年调研中“TKI医保报销政策”成为患者关注度最高的问题。近几年来，随着伊马替尼、达沙替尼和尼洛替尼先后进入全国医保目录和药物降价，CML患者所需承担的TKI治疗费用大幅下降。因此，在本次调研中，CML患者对“TKI医保报销政策”和“TKI药物价格”的关注度显著下降。

近年来，我国CML患者的TKI治疗结构发生了明显的改变，服用国产TKI和二代TKI的患者比例显著增长。2014年与2020年对我国CML患者TKI治疗状况的调研显示，服用国产仿制TKI的患者由7％增长至37％，服用二代TKI的患者由16％增长至35.4％[8]–[9]。本研究同样显示，与2015–2016年相比，本次调查受访者中服用国产TKI的患者由33.5％增长至42.0％，服用二代或三代TKI的患者由26.7％增长至44.6％。国内多项研究已证实，国产仿制与原研伊马替尼在CML慢性期患者治疗中的有效性和安全性差异无统计学意义[13]，并且由进口原研药转换为国产仿制药后，其良好的有效性、安全性及CML患者所获得相似的健康相关的生活质量（HRQoL）也已被临床研究所证实[14]–[15]。基于此，本调研中患者对“国产TKI仿制药质量”和“国产与进口TKI转换”这两个问题的关注度显著降低，表明国产仿制TKI药物质量已被越来越多的CML患者所信任，并取得了较为满意的疗效。尽管本次调研显示选择进口TKI的CML患者仍占多数，由于国产仿制药具有价格低廉、容易获取等优势，在未来预期会受到更多CML患者的欢迎。

耐药是影响CML患者获得不良结局的另一重要因素。在2015–2016年的调研中，半数以上患者对“TKI新药进展”问题给予了关注，表明当时仍有较多CML患者TKI治疗未能达到理想效果。近年来，数种二代、三代TKI相继上市或开展临床试验，解决了部分患者的治疗难题。因此，在本次调研中，患者对“TKI新药进展”的关注度较2015–2016年调研减少了一半。但仍有30％的患者关注“TKI耐药”，关注“TKI耐药”更多见于目前服用二代或三代TKI和治疗反应未达CCyR者。

本次调研中，半数以上CML患者关注“TKI不良反应及处理”，显著高于2015–2016年，而对“TKI远期疗效”的关注度显著下降。本次调研的受访者TKI治疗时间更长、获得CCyR和UMRD比例更高，推测可能与患者在TKI药物的治疗中取得了较为满意的治疗反应，不再过多为CML威胁生命问题而担忧，但经历长期用药中TKI相关不良反应的困扰，期待进一步提高生活质量相关。为进一步减轻患者的经济负担和TKI不良反应，“TKI停药”已成为CML患者所关注的重要问题。然而目前国外研究表明，在获得深层分子学反应的CML患者中，停药失败率近半数[16]–[18]，因此“TKI药物减量”已成为部分患者的治疗追求。本次调研中，多因素分析显示，女性、年龄大、学历≥本科、目前服用伊马替尼、进口TKI的受访者对“TKI药物减量”给予了更多的关注；我们既往研究发现，女性、老年、服用伊马替尼而非二代TKI与更高比例和更严重的患者报告的症状相关[19]；进口TKI价格相对更高；这些可以解释患者更希望TKI减量以期改善药物作用并减轻经济负担。

患者教育在CML疾病全程管理中发挥的作用不容小觑。近年来，在医务工作者的持续努力下，CML疾病相关知识的普及教育取得了成效。本次调研中，关注“CML基础知识”和“生育”[20]的受访者比例显著降低，而关注“基因监测”和“化验报告单的解读”的受访者比例显著提高，而且40％的患者关注“疾病危险度”和“生活中注意事项”，反映出CML患者在长期教育下，自我管理意识和对疾病认知意识的加强。

本次调研正值新型冠状病毒肺炎持续流行时期，近40％的CML患者关注了“新冠疫苗”问题。国外研究显示，恶性血液肿瘤患者的新型冠状病毒肺炎（COVID-19）感染率和死亡率高于健康人群[21]。2020年新冠疫情期间，本团队对CML患者心理健康状况的调查显示，患病人群相较健康人群具有更高的抑郁、焦虑和痛苦水平[22]。因此可以理解，CML患者更关注国家疫苗接种政策。目前，国家政策中标注白血病患者暂缓接种，仍需更多新冠疫苗在白血病患者中有效性和安全性的研究数据问世，以解决患者困惑。本次调研中，多因素分析显示，治疗反应≥CCyR、目前服用伊马替尼、进口TKI的CML患者对“新冠疫苗”给予了更多的关注，提示治疗反应较好的患者更多关注CML以外疾病的预防。

本研究存在以下缺陷：①通过网络平台发放电子问卷进行调研，老年、文化程度较低及不擅长使用互联网的患者未能纳入本研究，存在受访者选择偏倚；②受访者对疾病诊断、治疗用药和治疗反应的回忆信息未经专业人员证实，可能导致信息不准确；③TKI治疗费用的信息未被收集，无法准确评估患者经济负担的变化；④因2015–2016年调查为匿名调查，无法统计与本次实名调查受访者的重叠比例，可能影响患者关注问题变化的原因分析。

总之，本研究调查了2021年CML患者在TKI治疗过程中关注的问题和影响因素。本研究结果提示，临床医师应了解CML患者的需求，以患者为中心，针对具有不同社会人口学和疾病特征的患者设计合理的治疗策略，关注其身心健康，加强医患沟通，提高TKI总体治疗结局。
